# Neutrophils and anti-cancer immunity: a paradigm shift in cancer immunotherapy

**DOI:** 10.18632/oncoscience.378

**Published:** 2017-11-28

**Authors:** Rebecca Sturey, Akash Patnaik

**Affiliations:** Section of Hematology/Oncology, Department of Medicine, University of Chicago, Chicago, IL, USA

**Keywords:** neutrophils, cancer, immunity, immunotherapy

The idea of using the immune system to eradicate cancer dates back over a hundred years to William Coley injecting bacterial lysates to induce an anti-tumor response. Recent evidence has highlighted the pivotal role of the immune system in controlling cancer initiation, growth and progression. As malignancies acquire mutations which can manifest in altered peptides, tumors must sufficiently suppress or evade the immune system to survive.

Harnessing the immune system to attack cancers has been no easy task, and a variety of techniques have been deployed over the last several decades. These include introducing pro-inflammatory cytokines such as IL-2, IFN-α, or tumor antigens to trigger an immune response. Adoptive cellular therapies, which include priming antigen presenting cells outside the body and then re-introducing them back into the patient, are now FDA approved.[1] Researchers are also pioneering methods to use probiotics and oncolytic viruses to halt tumor progression.[2]

Several of our most recent discoveries have sparked a revolution in cancer immunotherapy. Over 18,000 articles have been published on the topic in just three years, as opposed to only 15,000 from 1900 to 1990. This dramatic increase is primarily due to the profoundly durable, complete responses to immune checkpoint blockade therapies which inhibit CTLA-4, PD1 or PDL1. By blocking these molecules, cytotoxic T-cells can effectively infiltrate tumors, ultimately resulting in tumor eradication. Unfortunately, only 10-30% of patients are capable of responding to checkpoint blockade therapies, and it has been a high research priority to develop mechanisms to increase the total number of responders.

In the midst of the myriad of research dominated by cytotoxic T cells and checkpoint blockade, “Cabozantinib Eradicates Advanced Murine Prostate Cancer by Activating Antitumor Innate Immunity” by Patnaik et. al. shifts focus to the innate immune system by demonstrating that a promiscuous receptor tyrosine kinase inhibitor can induce neutrophils to mediate an effective anti-tumor response and tumor regression. This regression occurred in an aggressive, highly representative, genetically engineered mouse model of prostate cancer, which recapitulates clinical features of poorly differentiated, treatment-refractory advanced prostate cancer.[3] Furthermore, since PTEN/p53 deficiency drives this model, the observations seen here may apply to a variety of different metastatic cancers.[4] In addition, the inducing agent, cabozantinib, is currently used to treat patients with thyroid or kidney cancer and can easily be repurposed to treat other types of cancer. The effects were strikingly immediate. Cabozantinib was able to entice neutrophils – first-responders of the immune system to infiltrate the tumor and eradicate the invasive prostatic adenocarcinoma in less than 72 hours. Mechanistically, this outcome was found to be independent of T cell or NK cell function, and could be abrogated by concomitant treatment with granulocyte depletion (dexamethasone), HMGB1 neutralization (3E8), or a CXCR4 inhibitor (plerixafor), thus affirming neutrophils as the key effectors. To our knowledge, this is the first demonstration of a tyrosine kinase inhibitor that modulates anti-tumor innate immunity, resulting in poorly differentiated invasive cancer eradication in a treatment-refractory PTEN/p53 deficient mouse model of prostate cancer.

While this strong chemotactic pull on neutrophils is necessary for their antitumor activity, it would be of particular interest to dissect how cabozantinib activates neutrophils to develop into pro-inflammatory, anti-tumor neutrophils (N1) instead of pro-tumorigenic neutrophils (N2).[5] This is particularly interesting considering that overall, neutrophils have been seen as predominantly pro-tumorigenic by supporting metastases formation and primary tumor growth.[6] Furthermore, a recent paper has noted neutrophil-mediated anti-tumor effects resulted in delayed growth and fewer metastases, but not a regression in tumor bulk.[7] Thus cabozantinib's activity profile may be used to generate a blueprint to determine whether other receptor tyrosine kinase inhibitors alone, or in combination, may elicit a similar anti-tumor immune response. Several of these tyrosine kinases may have to be inhibited in conjunction and at the right magnitude to elicit this immune effect. This would require a systems biology analysis to understand and replicate fully, and may be particularly difficult to tease out since cabozantinib can potently target c-MET, VEGFR, RET, KIT, AXL, FLT3, all of which are implicated in tumor growth and survival.

Collectively, the manuscript highlights the importance of tumor-infiltrating granulocytes within the tumor microenvironment, and provides a fascinating proof of concept of how kinase inhibitors can modulate the innate immune system against cancer. This occurred in a model where PI3K signaling is amplified due to PTEN loss, which has been shown to be inherently resistant to current immunotherapies.[8] Thus, this paper not only represents a paradigm shift in the field by showing that innate immune cells can independently provide an anti- tumor response, but also suggests that strategies to activate neutrophils against cancer may be one promising approach to overcome resistance to T cell based immunotherapies. This may be particularly relevant to castration resistant prostate cancer and pancreatic ductal adenocarcinoma, which have abnormally low response rates to checkpoint blockade therapies. In addition, activation of the innate immune system should prime the tumor microenvironment for adaptive immune activation by spewing inflammatory cytokines and tumor antigens. Thus, from a translational standpoint, this discovery paves the way for mechanistic proof-of-concept combination studies of cabozantinib and/ or other innate activating treatments with existing immune T cell checkpoint blockade inhibition.

In essence, the conclusions from this manuscript strike at the heart of a critical question: how do tyrosine kinase inhibitors, and other “targeted” therapies activate aspects of innate and/or adaptive anti-cancer immunity to equip the immune system to work at its highest potential against cancer.
